# Supplementary material for: Phosphoproteome modifications and cortical circuit dysfunction are linked to the early-stage progression of alpha-synuclein aggregation
Source: bioRxiv. 2025 Jun 5:2025.01.24.634820. Originally published 2025 Jan 25. Preprint. [Version 2] doi: 10.1101/2025.01.24.634820 (PMC11785254; doi:10.1101/2025.01.24.634820)
Supplement: 1 [file NIHPP2025.01.24.634820V2-supplement-1.pdf]

bioRxiv preprint doi: https://doi.org/10.1101/2025.01.24.634820; this version posted June 6, 2025. The copyright holder for this preprint (which was not certified by peer review) is the author/funder, who has granted bioRxiv a license to display the preprint in perpetuity. It is made available under aCC-BY 4.0 International license.

Supplementary Figure 1

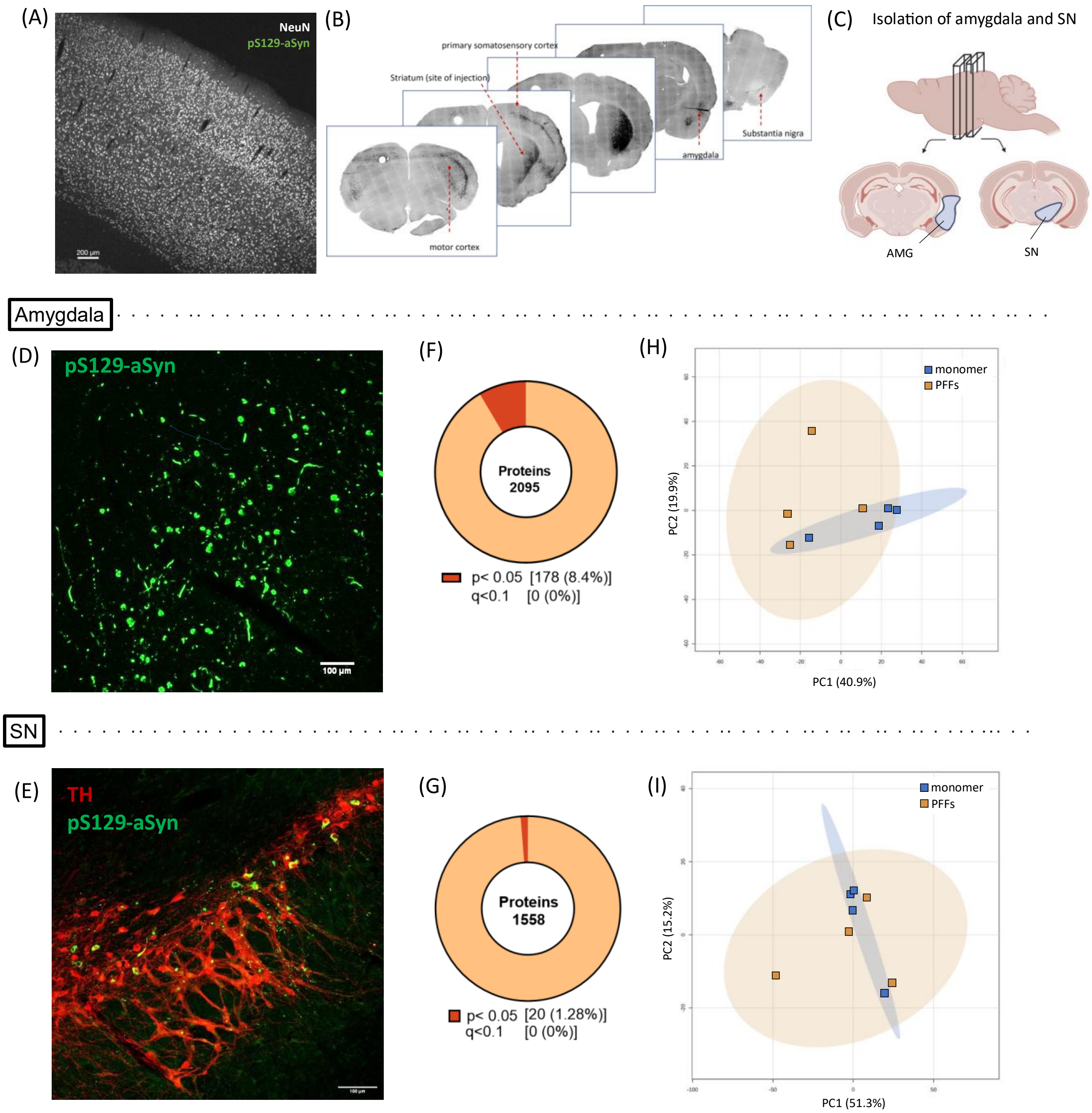

bioRxiv preprint doi: https://doi.org/10.1101/2025.01.24.634820; this version posted June 6, 2025. The copyright holder for this preprint (which was not certified by peer review) is the author/funder, who has granted bioRxiv a license to display the preprint in perpetuity. It is made available under aCC-BY 4.0 International license.

Supplementary Figure 1: Rats injected intrastriatally with aSyn PFFs do not show evidence of pronounced changes in the global proteome in the amygdala or SN.

(A) Image showing the absence of pS129-aSyn (81a) signal in a rat cortical section stained for the pan-neuronal marker (NeuN, white) 3 months after monomer injection in the striatum. Scale bar: 200 µm.

(B) Representative images of rat brain sections showing pS129-aSyn immunoreactivity in the cortex, striatum, amygdala, and SN 3 months after aSyn PFF injection in the striatum. Serial coronal brain sections were stained with the EP1536Y antibody.

(C) Schematic of serial brain sections used to collect amygdala (AMG) and SN samples for proteomic analysis (created using https://BioRender.com).

(D-E) Representative images of rat brain sections showing pS129-aSyn immunoreactivity (EP1536Y, green) in the amygdala (D) or SN (E) 3 months after aSyn PFF injection in the striatum. The nigral section in (E) was co-stained for tyrosine hydroxylase (TH) to demonstrate the presence of pS129-aSyn inclusions in nigral dopaminergic neurons (n = 3 animals). Scale bar: 100 µm.

(F-G) Pie chart representations of protein hits obtained via global proteomic analysis of homogenates prepared from rat amygdala (F) or SN (G) 3 months after intrastriatal injection with aSyn PFFs or monomer. The chart shows the percentage of hits with $p<0.05$ or $q<0.1$. Each hit was detected in ≥70% of samples in at least one experimental group.

(H-I) Graphs showing the results of unassigned/unsupervised PCA of the $\log_2$-transformed intensities of all protein hits identified in homogenates prepared from rat amygdala (H) or SN (I) as described in (F) and (G).

bioRxiv preprint doi: https://doi.org/10.1101/2025.01.24.634820; this version posted June 6, 2025. The copyright holder for this preprint (which was not certified by peer review) is the author/funder, who has granted bioRxiv a license to display the preprint in perpetuity. It is made available under aCC-BY 4.0 International license.

Supplementary Figure 2

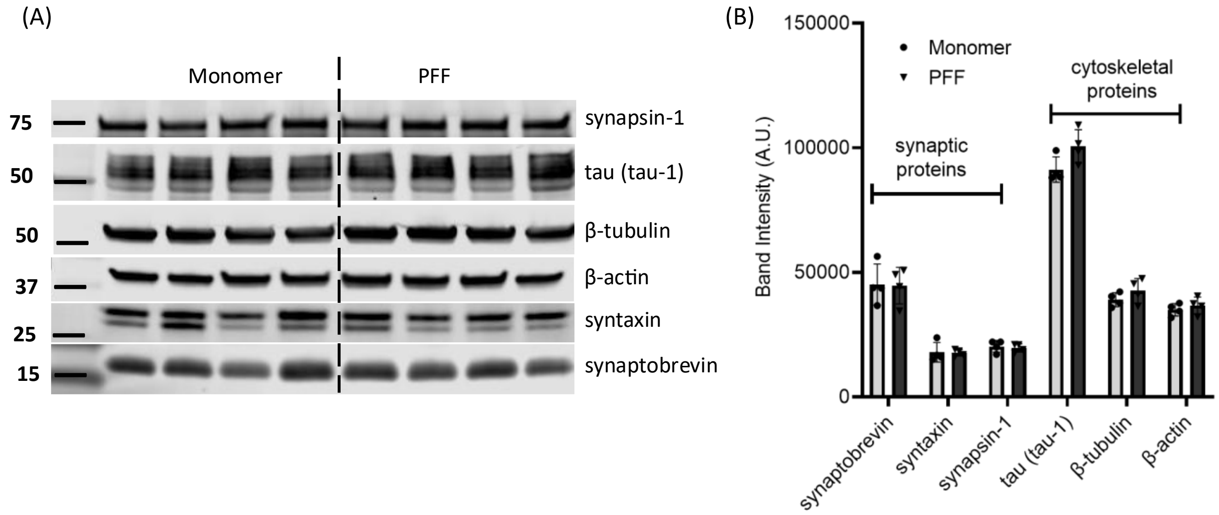

Supplementary Figure 2: Exposure to aSyn PFFs does not lead to changes in levels of synaptic or cytoskeletal proteins in rat sensorimotor cortex. (A) Image of Western blot loaded with homogenates prepared from rat sensorimotor cortex 3 months after intrastriatal injection with aSyn PFFs or monomer. The blot was probed with antibodies specific for the synaptic proteins synaptobrevin, syntaxin, and synapsin-1 or the cytoskeletal proteins tau, β-tubulin, and β-actin (n = 4 biological replicates). (B) Graph showing band intensities determined for the Western blot in panel A (mean ± SD). An unpaired t-test revealed no significant differences between the 'Monomer' and 'PFF' groups for each protein examined on the blot.

bioRxiv preprint doi: https://doi.org/10.1101/2025.01.24.634820; this version posted June 6, 2025. The copyright holder for this preprint (which was not certified by peer review) is the author/funder, who has granted bioRxiv a license to display the preprint in perpetuity. It is made available under aCC-BY 4.0 International license.

Supplementary Figure 3

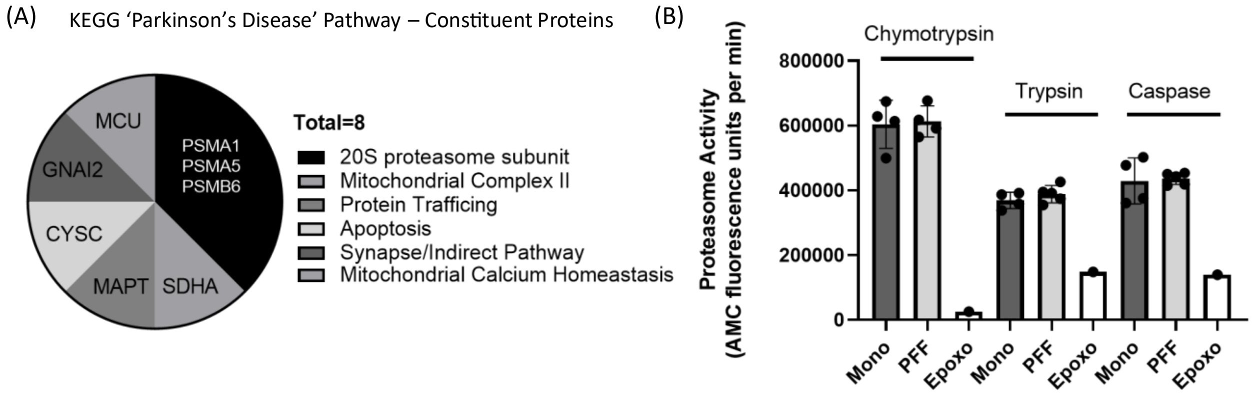

Supplementary Figure 3: Exposure to aSyn PFFs may lead to modest changes in the levels of PD-linked proteins (including 20S proteasome subunits) in rat sensorimotor cortex.

(A) KEGG pathway analysis of proteins from the global proteomics dataset with $p<0.05$ (Figure 1E) revealed 'Parkinson's disease' as the most significantly enriched pathway (log p = -5.17, log q = -3.21). The pie chart shows the 8 hit proteins from our dataset that constitute this pathway, classified into subgroups defined by different cellular functions. The largest subgroup ('20S proteasome subunit') contains 3 of the 8 protein hits. PSMA1, proteasome 20S subunit alpha 1; PSMA5, proteasome 20S subunit alpha 5; PSMB6, proteasome 20S subunit beta 6; SDHA, succinate dehydrogenase complex flavoprotein subunit A; MAPT, microtubule-associated protein tau; CYSC, cystatin C; GNAI2, guanine nucleotide-binding protein G(i) subunit alpha-2; MCU, mitochondrial calcium uniporter.

(B) Comparison of chymotryptic, tryptic, or caspase-like activity of the 20S proteasome in homogenates prepared from rat sensorimotor cortex 3 months after intrastriatal injection with aSyn PFFs or monomer. The graph shows enzyme activities expressed as rates of cleavage of the proteasome substrates SUC-LLVY-AMC (chymotrypsin), Boc-Leu-Arg-Arg-AMC (trypsin), and Ac-Nle-Pro-Nle-Asp-AMC (caspase-like). Bars labeled 'Epoxo' correspond to homogenates prepared from control rats, pretreated with the proteasome inhibitor epoxomicin (n = 1; final concentration, 5 µM) for 20 min prior to measuring proteasome activity (mean ± SD, n = 4 or 5 biological replicates). A one-way ANOVA revealed no significant differences between the 'Mono' and 'PFF' groups for each of the 3 enzymatic activities.

bioRxiv preprint doi: https://doi.org/10.1101/2025.01.24.634820; this version posted June 6, 2025. The copyright holder for this preprint (which was not certified by peer review) is the author/funder, who has granted bioRxiv a license to display the preprint in perpetuity. It is made available under aCC-BY 4.0 International license.

Supplementary Figure 4

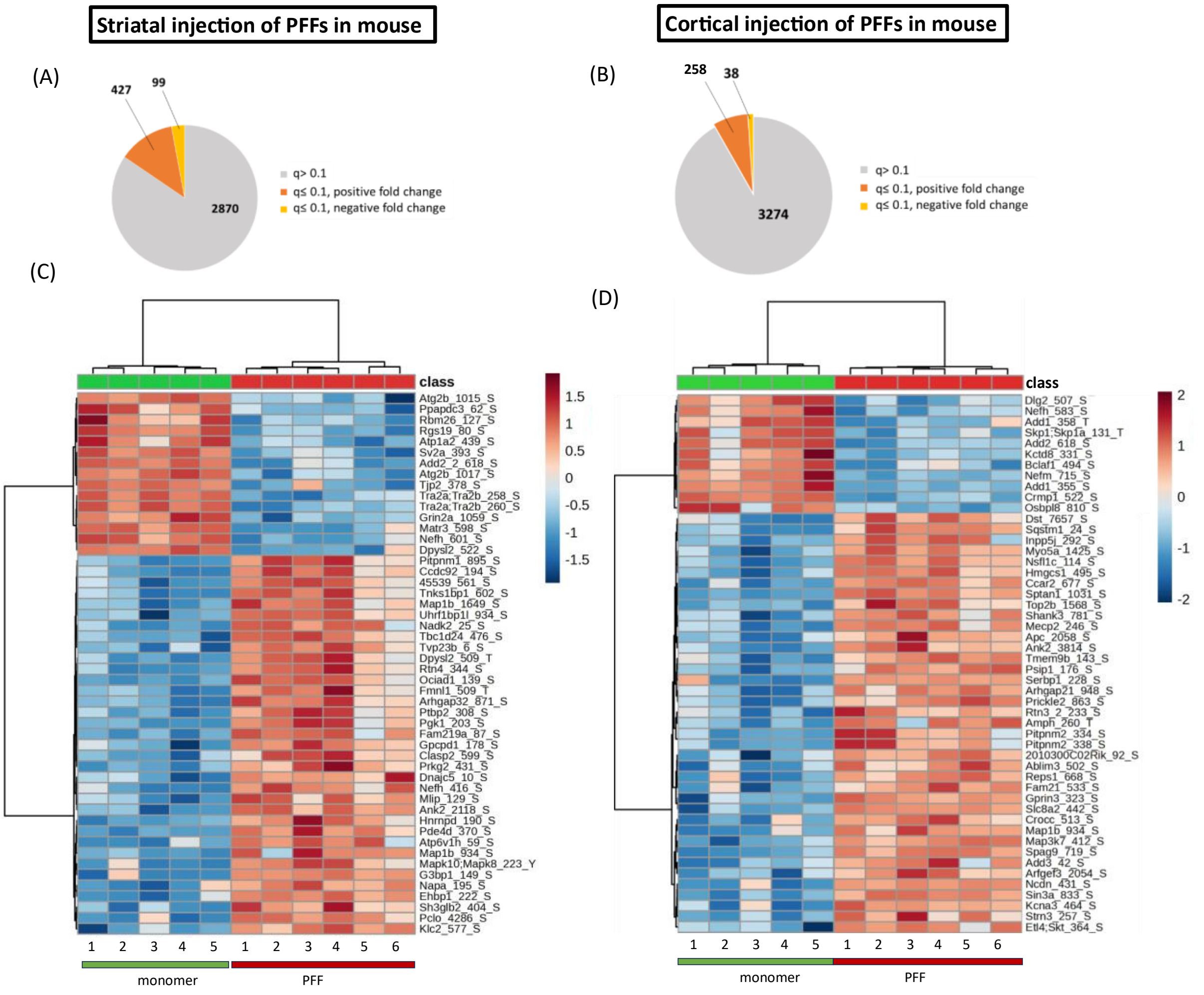

Supplementary Figure 4: Extended analysis of phosphoproteomic changes in the sensorimotor cortex of mice following intrastriatal or intracortical injections of aSyn PFFs.

(A, B) Pie chart representations of phosphosite hits obtained via phosphoproteomic analysis of homogenates prepared from mouse sensorimotor cortex 3 months after injection with aSyn PFFs or monomer in the striatum (A) or cortex (B). Each chart shows the percentage of hits with $q<0.1$ with a positive or negative fold change (orange or yellow, respectively). Each hit was detected in ≥70% of samples in at least one experimental group.

(C, D) Clustered heatmaps showing log_2-transformed intensities of the top 50 up- or down-regulated phosphosites (i.e., phosphosites with the lowest q-values) in cortical homogenates from mice injected with aSyn PFFs or monomer in the striatum (C) or cortex (D), as described in (A) and (B). Red and blue colors correspond to an increase or decrease (respectively) in phosphosite levels in mice injected with PFFs versus monomer, and the color intensity represents the Z-score-normalized log_2(intensity) value. Peptide names are listed as ‘protein name_phosphoresidue number.’

bioRxiv preprint doi: https://doi.org/10.1101/2025.01.24.634820; this version posted June 6, 2025. The copyright holder for this preprint (which was not certified by peer review) is the author/funder, who has granted bioRxiv a license to display the preprint in perpetuity. It is made available under aCC-BY 4.0 International license.

Supplementary Figure 5

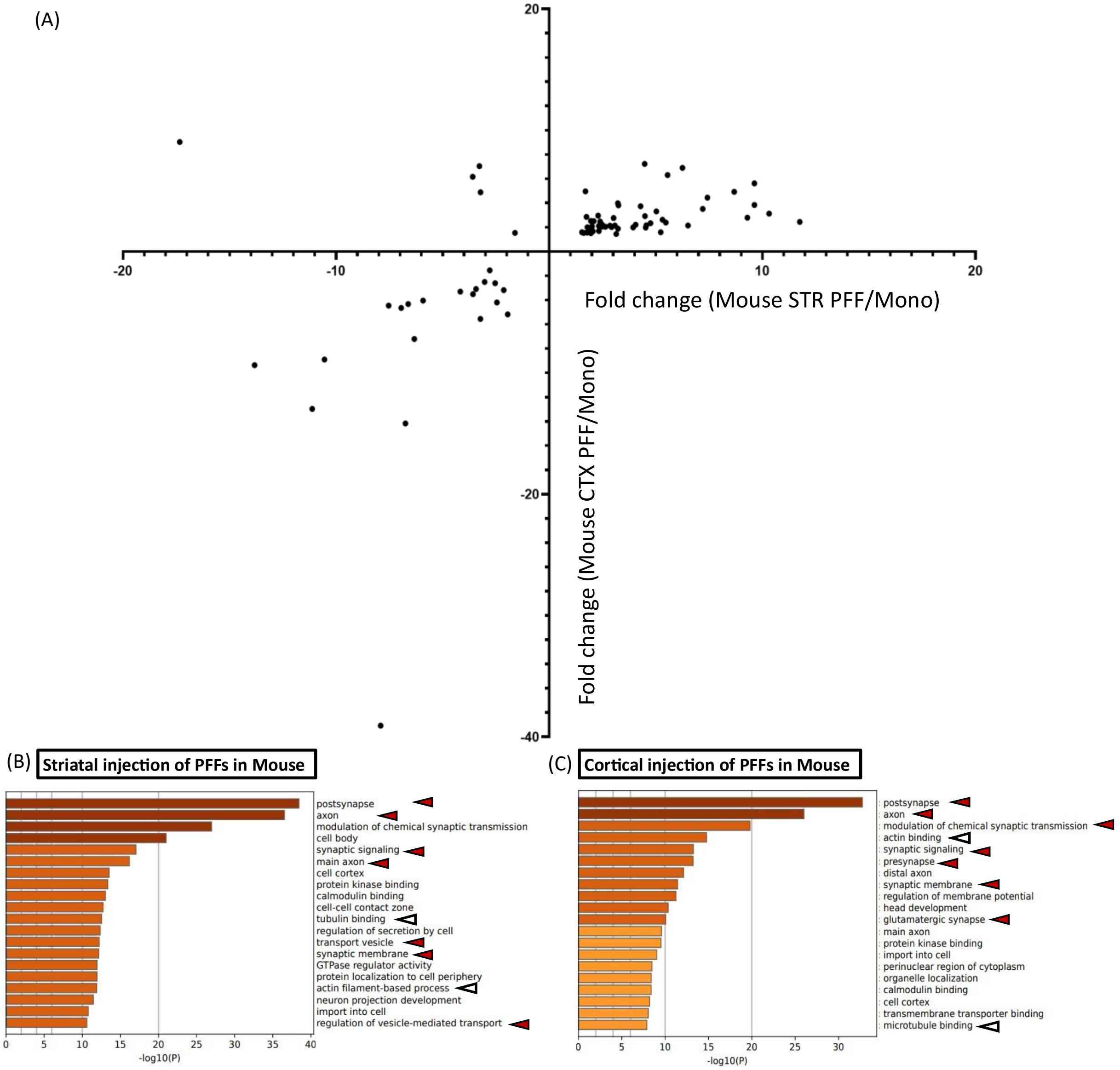

Supplementary Figure 5: Mice injected with aSyn PFFs in the striatum or cortex show evidence of altered synaptic function and cytoskeletal organization. (A) Graph depicting fold-change values of significantly altered phosphosites in the sensorimotor cortex of mice injected intrastriatally (STR) or intracortically (CTX) with aSyn PFFs versus monomer. (B, C) Charts showing enriched GO terms in the 'cellular localization' category that represent groups of phosphoproteins in mouse sensorimotor cortex impacted by PFF administration in the striatum (B) or cortex (C), 3 months post-injection. GO analysis was carried out on proteins containing phosphosites with q<0.1 and a fold change of ≥2 (Supplementary Figure 4A,B). Arrowheads highlight items relevant to synaptic transmission (red) or the cytoskeleton (white).

bioRxiv preprint doi: https://doi.org/10.1101/2025.01.24.634820; this version posted June 6, 2025. The copyright holder for this preprint (which was not certified by peer review) is the author/funder, who has granted bioRxiv a license to display the preprint in perpetuity. It is made available under aCC-BY 4.0 International license.

Supplementary Figure 6

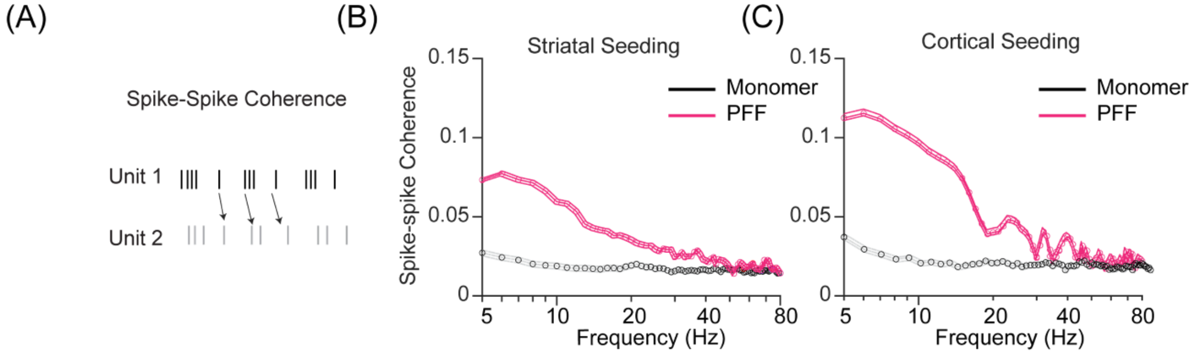

Supplementary Figure 6: Mice injected with aSyn PFFs in the striatum or cortex show evidence of altered network connectivity. (A) Schematic of spike-spike coherence where the coherence of two representative units is calculated based on fluctuations of spike rates (see 'Experimental Methods'). (B, C) Graphs showing frequency-resolved paired spike coherence data recorded in mouse sensorimotor cortex 3 months after injection with aSyn PFFs or monomer in the striatum (B) or cortex (C).

bioRxiv preprint doi: https://doi.org/10.1101/2025.01.24.634820; this version posted June 6, 2025. The copyright holder for this preprint (which was not certified by peer review) is the author/funder, who has granted bioRxiv a license to display the preprint in perpetuity. It is made available under aCC-BY 4.0 International license.

Supplementary Figure 7

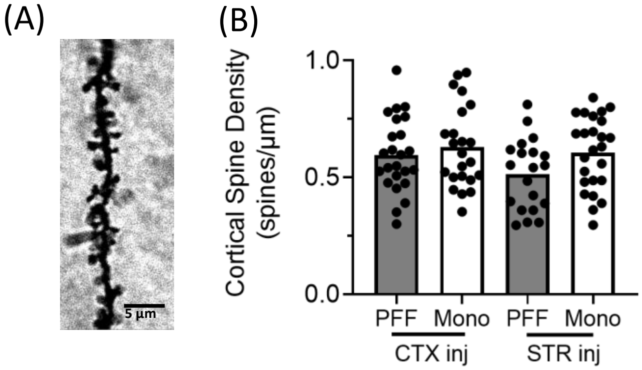

Supplementary Figure 7: Seeded aSyn aggregation does not lead to a change in cortical spine density in our mouse PFF model. (A) Representative image of a dendritic section from layer II/III of mouse sensorimotor cortex 3 months after intrastriatal aSyn PFF injection (scale bar: 5 µm). (B) Graph showing the spine density in sections prepared from mouse sensorimotor cortex 3 months after intracortical (CTX) or intrastriatal (STR) injection with aSyn PFFs or monomer. Five dendritic sections were analyzed per mouse, and 4 to 5 mice were analyzed per group. A one-way ANOVA revealed no significant differences among any of the groups.

bioRxiv preprint doi: https://doi.org/10.1101/2025.01.24.634820; this version posted June 6, 2025. The copyright holder for this preprint (which was not certified by peer review) is the author/funder, who has granted bioRxiv a license to display the preprint in perpetuity. It is made available under aCC-BY 4.0 International license.

Supplementary Figure 8

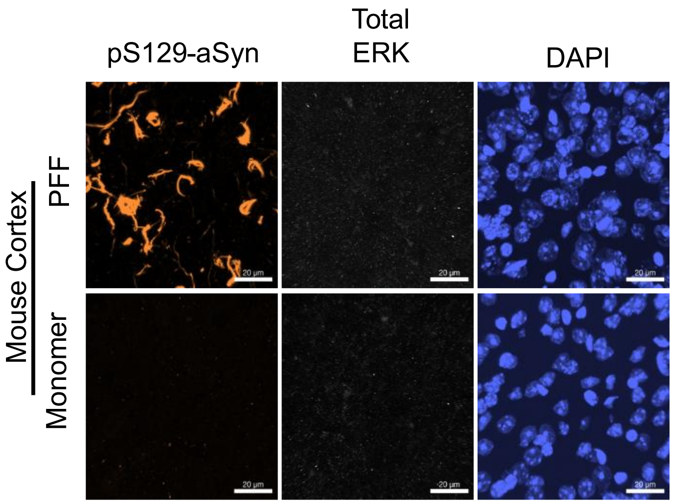

Supplementary Figure 8: Mice show no evidence of total ERK up-regulation in aSyn aggregate-containing neurons after intrastriatal aSyn PFF injection. Representative images show no detectable difference in total ERK levels in the sensorimotor cortex of mice injected with aSyn PFFs versus monomer 3 months post-injection (n = 4).

bioRxiv preprint doi: https://doi.org/10.1101/2025.01.24.634820; this version posted June 6, 2025. The copyright holder for this preprint (which was not certified by peer review) is the author/funder, who has granted bioRxiv a license to display the preprint in perpetuity. It is made available under aCC-BY 4.0 International license.

Supplementary Figure 9

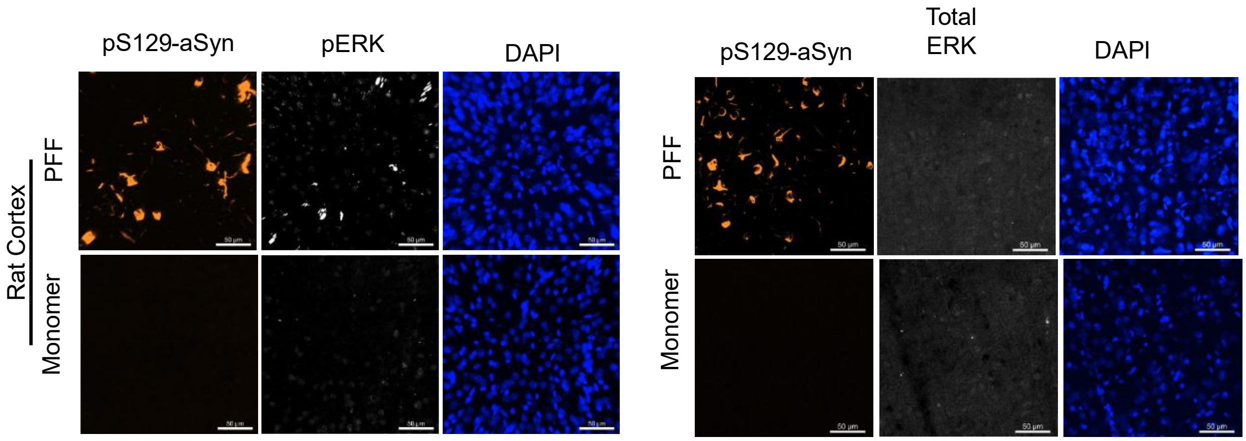

Supplementary Figure 9: Rats injected with aSyn PFFs exhibit an increase in phospho-ERK levels. (Left) Representative images show increased phospho-ERK (pERK) levels in the sensorimotor cortex of rats injected intrastriatally with aSyn PFFs versus monomer 3 months post-injection (n = 3), confirming ERK pathway activation. (Right) No detectable difference in total ERK levels was observed.
